# Intravenous Medication Administration Errors in Hospitalised Patients: An Updated Systematic Review

**DOI:** 10.1111/jep.70167

**Published:** 2025-06-22

**Authors:** Sutha Rajakumar, Retha Rajah, Subramaniam Thanimalai, Fadzlin binti Mohd Mokhtar, Dinesh Sangarran Ramachandram

**Affiliations:** ^1^ Department of Pharmacy Hospital Seberang Jaya, Jln Tun Hussein Onn, Seberang Jaya Pulau Pinang Malaysia; ^2^ Jeffrey Cheah School of Medicine and Health Sciences, Monash University Malaysia Subang Jaya Selangor Malaysia; ^3^ Department of Pharmacy Hospital Sultanah Bahiyah Alor Setar Kedah Malaysia; ^4^ Department of Pharmacy Sultan Idris Shah Hospital Kajang Selangor Malaysia; ^5^ School of Pharmacy, Monash University Malaysia, Jalan Lagoon Selatan, Bandar Sunway Subang Jaya Selangor Malaysia

**Keywords:** hospitalised, in‐patients, intravenous, medication administration, medication errors

## Abstract

**Background:**

Administering intravenous (IV) drugs carries a high risk of adverse effects due to their direct entry into circulation. Identifying the prevalence and types of IV administration errors and the drugs involved is crucial for implementing effective interventions to reduce such errors.

**Aim:**

This systematic review aimed to examine and synthesise the available articles on medication errors involving IV administration in hospitalised patients.

**Methods:**

A comprehensive search was conducted using electronic databases, including PubMed, Ovid Medline, and CINAHL. The search was performed without time limitation up to July 2023. However, only articles published in English and human subjects were included. The quality of the studies was appraised using the Newcastle‐Ottawa quality assessment scale (NOS). This systematic review was registered with PROSPERO (CRD42023469352).

**Result:**

Database searches yielded 2177 articles; after duplicate removal, 1717 underwent title and abstract screening, and 23 were included after full‐text review. The studies were from 12 countries, and the multicentre study included countries from Europe, Africa, the Americas, Asia, and Australia. The majority of the studies were conducted in either teaching hospitals (*n* = 11) or university‐affiliated hospitals (*n* = 7), with most involving direct observation (*n* = 21). IV administration errors exhibit a broad prevalence range of 5.0%−62.9%, involving various types such as wrong diluent, dose, route, rate, technique, omission, and timing. Studies lack uniformity in reporting, with some not specifying prevalence. The highest prevalence of specific errors varies across settings.

**Conclusion:**

Our review highlights that IV medication error rates vary based on study design, setting, and population. Standardised definitions, reporting procedures, and reliable tracking methods are needed. Human factors, system issues, and environmental stressors influence medication errors. Future research must improve our understanding and address these factors to enhance patient safety and healthcare quality.

## 1 Introduction

Medication‐related harm affects 1 in every 30 patients seeking treatment in healthcare facilities, with some harm being severe or life‐threatening (WHO Patient Safety). The US Institute for Safe Medication Practice (ISMO) has reported that more than half of the medication errors were associated with medication administered intravenously [1]. The ECRI Institute also stated that IV medication errors are the number one threat to patient safety [2]. The intravenous (IV) route of drug administration is widely used in hospitals due to its immediate effects and high bioavailability. However, IV medication is complex to prepare and administer, with many in the high‐alert medication category that can cause significant patient harm if used in error.

**Figure 1:**
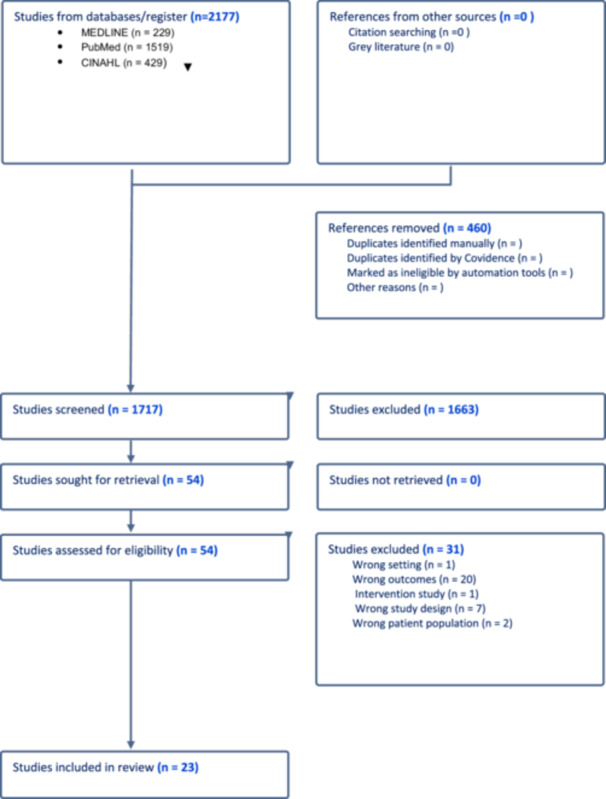
Prisma flow chart.

Several prior systematic reviews have dealt with administration errors in IV medications [3, 4, 5]. However, the heterogeneity and the outcome measures of the included studies obscure the true nature of the problem. In an earlier systematic review of 9 studies on IV medication, the findings reported that one of the highest errors was medication administration at 21.7% [3]. However, this review had a high degree of heterogeneity as it involved 12 stages of preparation, administration, and documentation of the IV medication. Another systematic review of 11 studies investigating the systemic causes of IV medication errors in hospitals showed the most reported errors were in the administration stage (six studies) [4]. In a recent systematic review conducted only on UK studies specific to geographical areas that may limit generalisability [5], the error rate reported was highest during the administration phase, with a mean rate of 32.1% of errors [5]. Similar to other previously mentioned systematic reviews, this review also focused on different stages of IV medication administration, including prescribing, preparation, and administration (Figure 1).

IV administration error may be the direct cause of human error; however, frequently, the error is supported by a broken system with insufficient backup to catch errors. To draft the intervention to minimise errors in IV medication administration, the depth of the error and the type of errors specific to the administration must be determined. This systematic review aimed to critically appraise, synthesize, and present the evidence amongst hospitalised patients.

## 2 Materials and Methods

### 2.1 Search Strategy

The systematic review protocol was developed following the Preferred Reporting Items for Systematic Review and Meta‐Analysis (PRISMA) guidelines and registered with the International Prospective Register of Systematic Reviews (PROSPERO) with registration number CRD42023469352. The literature search was conducted in four electronic databases, such as PubMed. Ovid EMBASE, CINAHL, and Scopus. The search was conducted between August 2023 and September 2023. The search strategy used the following keywords connected by Boolean operators OR and AND, (medication OR drug) AND (administration AND error) AND (ward OR hospital OR inpatient). For each database, the search strategy was specified in Supporting Information S1: Appendix 1.

### 2.2 Inclusion and Exclusion Criteria

Inclusion criteria comprised the following: (1) articles published in the English language up to July 2023; (2) studies that contained a description of IV medication administration error in inpatient settings or hospitalised patients; (3) Prospective, cross‐sectional, observational, or interventional before‐after studies were included in our analysis. For interventional before‐after studies, only the administration error rate calculated in the period before the intervention was considered. Exclusion criteria comprised (1) the outpatient setting or primary care facilities due to the assumption of the difference in medication handling and flow and (2) qualitative studies, editorials, reviews, notes, comments, conference proceedings, and letters.

### 2.3 Selection of Studies

Any duplication was removed after the articles were identified from the four databases. Two reviewers (R.R. and S.R.) independently screened all the studies for title and abstract in Covidence based on the inclusion and exclusion criteria. After discussion, discreparncies were resolved by consensus among the two reviewers (R.R. and S.R.). Many studies were excluded to ensure methodological rigor and relevance to IV medication errors in hospitalised patients. Studies were excluded if they did not specifically focus on IV medication errors, lacked primary data (e.g., narrative reviews, opinion pieces), had methodological limitations (e.g., unclear study design, small sample sizes, or lacked denominator data for calculating error rates), were conference abstracts or inaccessible full texts, or were conducted outside the defined time frame or in non‐hospitalised settings. These criteria were applied to maintain the validity and reliability of the findings.

### 2.4 Data Extraction

Data extraction was performed using a standardised form on a Microsoft Excel spreadsheet to extract the authors' names, year of publication, country of origin, study setting and population, study type, study design, and sampling method, the definition of IV administration error, the numerator, and denominator for the IV medication administration error, type of errors, drug involved in the error. For studies reporting IV medication errors, data extraction focused on both numerators (number of errors) and denominators (total IV administrations, doses, or patients exposed). However, some studies did not explicitly define their denominator, leading to variability in error rate calculations. Studies were categorised based on denominator clarity to address this, and findings were synthesised accordingly. Where possible, studies that provided relative measures (e.g., errors per 1000 administrations) were prioritised for comparison.

### 2.5 Risk of Bias Assessment

Two reviewers (S.T. and D.S.) conducted the risk of bias assessment using the Newcastle Ottawa quality assessment scale (NOS). The scale assessed the domain of selection, comparability, and outcome/exposure. At the end of the assessment, according to the criteria met by each study, the score was categorised as either high quality (score more than 7), moderate quality (score of 5−7), or low quality (score less than 5). Discussion resolved disagreements between the reviewers, and the third reviewer (F.M.M.) involved when necessary.

## 3 Results

### 3.1 Selection of Studies

A total of 2177 articles from the initial search underwent duplicate removal, resulting in 460 articles being removed. The remaining 1717 were screened for titles and abstracts, and 1663 articles were excluded. In the full‐text screening stage of 54 articles, 31 articles were excluded based on the following exclusion criteria: —different setting (*n* = 1), different outcomes (*n* = 20), intervention study (*n* = 1), different study design (*n* = 7) and different patient population (*n* = 2). The remaining 23 articles were retrieved and included in this review.

### 3.2 Characteristics of Included Studies

The characteristics of the 23 studies included are described in Table 1.

**Table 1:** Characteristics of the included studies.

Study, author, year of publication	Setting	Duration	Design	Study setting	Study population	Study design, sampling	IV medication error definition	Area of error	Observer; Observed
Cousins et al. [7]	UK, Germany and France	5 weeks to 3 months	Audit	Six hospital departments	Adults (general ward and intensive care)	Direct observation, Convenience sampling	A deviation in the preparation or administration of a drug from the doctor's prescription, the hospital's intravenous policy, or the manufacturer's instructions	Preparation Administration	Single (pharmacy technician/pharmacy student); nurses NS
De Castro et al. [10]	Brazil	2 months	Cross‐sectional, prospective	Teaching hospital	Adults (ICU)	Direct observation, Convenience sampling	Medication administration errors considered were dose omissions or omissions caused by failures in drug scheduling	Administration	NS
Ding et al. [9]	China	2 weeks	NS	Teaching hospital	Adults (surgery wards)	Direct observation, Convenience sampling	Any ingredient observed that was mixed in the IV bag and administered to the patients was different from the interpretable physician's orders written on the patient charts.	Administration	Single (a PhD candidate); nurses Trained and certified
Fahimi et al. [11]	Iran	1 year	NS	University‐affiliated centre	Adults (medical, surgical ICU, oncology, CCU)	Direct observation, NS	NS	Preparation Administration	Single (pharmacist); nurses Trained
Fahimi et al. [12]	Iran	3 months	NS	Teaching hospital	Adult (ICU)	Direct observation, Randomly selected	Any deviation in preparation and method of administration from the manufacturer's instructions	Preparation Administration	Single (pharmacist); nurses Trained
Fekadu et al. [33]	Ethiopia	2 months	Cross‐sectional	University affiliated centre	Adults (medical, surgical, gynaecology)	Direct observation, Simple random sampling	A deviation from the physicians' order as written on the patient's chart	Administration	Two (undergraduate pharmacists); NS
Hoefel et al. [13]	Brazil	7 months	NS	University affiliated hospital	Adults (ICU, Surgery, clinical inoatient)	Direct observation, NS	NS	NS	Five observers; assistants and nursing technicians trained
Nguyen et al. [14]	Vietnam	4 months	Prospective	Public hospital	Adults (ICU, post‐surgery wards, general medical, trauma unit)	Direct observation, Convenience sampling	Deviation in preparation and administration of oral or intravenous medications from the doctor's preparations, the hospital policies, or product instructions	Preparation Administration	Four observers (pharmacy students); nurses Trained, data checked for validity
Basil et al. [35]	Malaysia	1 month	Cross‐sectional	Public hospital	Adult and paediatric (12 wards)	Direct observation, Random sampling	Any deviations in the dose administered from the prescribed medication on the patient's medication chart, standard hospital policies or manufactures' instructions	Preparation Administration	Two observers (pharmacists); nurses Checked and validated, clinical outcome and factors associated with IV ME
Anselmi et al. [15]	Brazil	3 months	Cross‐sectional	General hospital	NS	Direct observation, Convenience sampling	A dose prepared and/or administered by nursing personnel is different than that prescribed by the physician and on the patient's record	Preparation Administration	Two‐six observers (nurses); nurses Training; validated
Dhamija et al. [16]	India	8 months	Audit	NS	Paediatric (Oncology)	Direct observation and self‐reporting, Convenience sampling	A preventable mistake in drug administration because of errors originating in ordering, dispensing, or administering chemotherapy	Ordering Charting Administration	Single; nurses NS
Mendes et al. [20]	Brazil	6 months	Cross‐sectional	University‐affiliated hospital	Adult patients (Emergency department)	Direct observation, Convenience sampling	NS	Preparation Administration Dispensing	Single observer; nurses NS
Sheu et al. [17]	NS	NS	NS	Teaching, local and regional hospitals	Medical and surgical	NS, Snowball sampling	NS	NS	NS NS
Sorgini et al. [18]	Canada	NS	Prospective	University‐affiliated hospital	Medico‐surgical ICU	Direct observation, NS	An infusion error was defined as any error of omission or Commission in the context of IV drug therapy that harmed or could have harmed a patient	Administration Documentation Prescription	4 observers (pharmacists); nurses NS
Prot et al. [19]	France	12 months	NS	Teaching hospital	Paediatric	Direct observation, NS	Any discrepancy between printed or handwritten physicians” order and drug delivery to the patient, in keeping with the classic	NS	12 observers (fifth‐year pharmacy students); nurses Trained Data validated
Chua et al. [21]	Malaysia	3 months	Prospective	Teaching hospital	Haematology wards	Direct observation, Convenience sampling	Discrepancy between drug therapy received by the patient and the intended by the prescriber or according to standard hospital policies and procedures	Administration	Single observer; ward staff Validated Clinical outcomes
Taxis and Barber [22]	United Kingdom	76 days	Prospective	Teaching and non‐teaching hospitals	Paediatric and adult (10 wards)	Distinguished observation, Purposive sampling	A deviation in the preparation or administration of a drug from the doctor's prescription, the hospital's intravenous policy, or the manufacturer's instructions	Preparation Administration	Single observer; nurses Clinical importance Trained
Barber and Taxis [23]	Germany	1 month	NS	Non‐university hospital	Adult (surgical and surgical ICU)	Direct observation, Convenience sampling	A deviation in the preparation or administration of a drug from the doctor's prescription, the hospital's intravenous policy, or the manufacturer's instructions	Preparation. Administration	Single observer; nurses Trained
Hermanspann et al. [24]	Germany	2 months	NS	University‐affiliated hospital	Paediatric (NICU and PICU) Adult (1CU)	Direct observation, Convenience sampling	Any instance in which the preparation or administration of injectable drugs varied from the physician's prescription, the SMPC of the drug, hospital policies and procedures, or the good manufacturing practice	Preparation Administration	2 observers (a clinical pharmacist and medical student); nurses Trained
Vijaya Kumar et al. [25]	India	4 months	Prospective	Private hospital	All wards except ICU and Oncology	Structured case report and direct observation, NS	Any error of using wrong rate or dilution in the context of administering medications intravenously	NS	NS NS
Westbrooke et al. [28]	Australia	6 months	NS	Teaching hospital	6 wards	Direct observation, NS	A deviation in the preparation or administration of a medicine from a doctor's legal prescription, reference books, or the manufacturer's instructions	Preparation Administration	3 observers (nurses and doctors); nurses Trained validated
Ong and Subasyini [26]	Malaysia	4 months	Prospective	Public hospitals	34 wards	Direct observation, Convenience sampling	A deviation in the preparation or administration of a medicine from a doctor's legal preparation, reference books, or the manufacturer's instruction	Preparation Administration	Single observer; nurses NS
Wirtz [27]	Germany	4 months	NS	Teaching hospitals	NS	Distinguished observation, Convenience sampling	Any deviation of preparation or administration of an IV dose from the original prescription or any act in the preparation or administration, which deviated from the manufacturer's instructions or the hospital drug policy	Preparation Administration	Single observer (pharmacist); nurses and doctors NS

Abbreviation: NS, not specified.

#### 3.2.1 Country and Year of Publication

Nine studies originated from Asia countries, including Malaysia (*n* = 3), India (*n* = 2), Iran (*n* = 2), China (*n* = 1) and Vietnam (*n* = 1). The highest number of studies was conducted in the following countries; four were conducted in Brazil, and three more were conducted in Germany. One multi‐centre study was conducted in the UK, Germany, and France. The remaining articles were from the United Kingdom (*n* = 1), Canada (*n* = 1), Ethiopia (*n* = 1) and Australia (*n* = 1). The studies were published between 2003 and 2019 (Table 1).

#### 3.2.2 Study Setting and Population

Most of the studies were conducted in either teaching hospitals (*n* = 12) or university‐affiliated hospitals (*n* = 6), and some were in general hospitals (*n* = 9). The wards/units for the studies generally involved adult wards (*n* = 18), and other studies involved paediatric wards (*n* = 5). The studies ranged from 2 weeks to 1 year (Refer to Table 1).

#### 3.2.3 Study Design and Sampling

Almost half of the studies (*n* = 10) included have no data on the study design. Four studies were cross‐sectional, six were prospective, and one was cross‐sectional, prospective study design. Two other studies mentioned the study design as an audit. The majority of the studies (*n* = 18) used the direct observation method. The combination of methods, including observation with self‐reporting in one study or stratified case report with observation, was used in two studies, and disguised direct observation was performed in two studies. Eighteen studies involved convenience sampling, while the rest used random sampling (*n* = 2), snowballing sampling (*n* = 1), and purposive sampling (*n* = 1) (Refer to Table 1).

#### 3.2.4 Observers and Validation of IV Medication Administration Error

Almost half of the studies involved a single observer (*n* = 12), while the eight others involved two or more observers. Eleven of those studies involving observers reported that the observer/s were either trained or went through training to observe and document the error. Most (*n* = 9) of the observer/s consist of pharmacists, undergraduate pharmacy students, or pharmacy doctorate students. The observed personnel mostly involved nurses (*n* = 17); one study observed both nurses and doctors. Only six studies had data on the validation of the collected data. One study specifically calculated inter‐rater reliability for two observers, and four studies described the data collected were checked by senior team members or supervisors (Refer to Table 1).

#### 3.2.5 Definition of IV Administration Error

Almost three‐quarters (*n* = 19) of the studies had a definition of IV medication error. The definition differs based on the process where the IV medication error occurred, whether in the preparation and administration stage, administration phase alone, or involving other stages like ordering and dispensing. Most of the studies (*n* = 13) reported on IV medication preparation and administration errors, while some studies only had data on IV medication administration errors (*n* = 5) (Refer to Table 1).

#### 3.2.6 Prevalence of IV Administration Error

IV administration error ranges widely from 5.0% to 62.9% [8, 9, 10, 11, 12, 13, 14, 15, 16, 20, 21, 22, 23, 24, 25, 28, 29, 30, 31, 32, 33, 34]. The types of IV administration errors are wrong diluent or dilution error, wrong dose or infusion volume, wrong route, wrong administration rate and technique, dose omission or unordered doses, and wrong time [6]. Several papers on IV administration did not report the prevalence of IV administration error [7, 10, 12, 13]. Some studies stated the number of errors in the setting; however, the prevalence was unavailable as the total number of observations or denominator was not specified [16, 20, 21, 22, 23, 24, 25, 28]. The type of IV administration error with the highest prevalence differs from one setting to another. For example, Cousins et al. have the highest prevalence of wrong diluent (28.6%), Ding et al. mentioned wrong dose (5.6%), Fahimi et al. on inappropriate administration rate (35.3%), Hoefel et al. on wrong time (34.9%), and Nguyen et al. on wrong administration technique (47.6%) [7, 9, 12, 13, 14].

### 3.3 Quality of Studies

The quality assessment of the 23 articles using the Newcastle‐Ottawa Scale (NOS) revealed a range of scores from 5 to 8 stars. The mean score was 6.61, and the median score was 7, indicating that the overall quality of the studies was relatively high. Most studies scored well in the selection and comparability domains, with a few receiving lower scores due to outcome/exposure assessment issues (Tables 2 and 3).

**Table 2:** Characteristics of study population and ascertainment of outcome.

Author	Name of drug	Subject	IV medication error	IV administration error	Type of IV administration error
Cousins et al. [7]	All intravenous drug preparation and administration	IV medication doses		NS	Wrong diluent (28.6%) Wrong dose/infusion volume (1.6%) Wrong route (1.0%) Wrong administration rate (21.4%) Wrong time (7.6%)
De Castro et al. [10]	Medications were classified according to the Anatomical Therapeutic Chemical Code ‐ Anti Infective mediations were analysed	IV medication doses		NS	Dose omission (4.3%)
Ding et al. [9]	IV preparation TPN and non‐TPN	IV medication doses		12.8%	Dose omission (2.3%) Wrong dose (5.6%) Wrong time (4.1%) Unordered dose (0.5%) Extra dose (0.3%)
Fahimi et al. [11]	21 IV medications	IV medication doses		NS	Inappropriate administration rate (35.3%) Drug‐drug interaction – (0.9%) Physiochemical incompatibility – (0.44%)
Fekadu et al. [33]	2 or more IV administered	IV medication intervention		46.1%	Dose omission (95.8%), Wrong dose (4.2%)
Hoefel et al. [13]	Cefepime	IV medication doses		NS	Wrong dose (11%) Wrong administration rate (27.7%) Wrong time (34.9%) Wrong administration technique (21.4%)
Nguyen et al. [14]	Intravenously via continuous infusion (i.e., excluding intermittently administered intravenous medications) only on the day of data collection	IV medication doses	39.1%	NS	Dose omission (5.1%) Wrong drug (2.7%) Wrong dose (3.9%) Wrong administration technique (51.3%)
Basil et al. [35]	Intravenous preparation	IV medication doses	85.0%	62.5%	Dose omission (2.0%) Wrong time (12.7%) Extra dose (0.3%) Wrong administration technique (47.6%)
Anselmi et al. [15]	Intravenous medication	IV medication doses	NS	5.0%	Dose omission (0.8%; 1.1%; 3.4%) Wrong dose (1.3%; 2.2%; 5.7%) Wrong drug (0.1%; 0; 0.2%)
Dhamija et al. [16]				205	Nurse administration with error = 10/23
Mendes et al. [20]	IV ‐ antimicrobials, non‐opioid analgesics, anti‐inflammatory agents, anti‐emetics, opioid analgesics, antacids, antiarrhythmic agents, diuretics, anticonvulsants, vasodilators, antispasmodics, cardiotonic agents, splenic vasodilators, antidiabetics, vasopressors, vitamins, and bone catabolism inhibitors			303 observations of preparation and administration of IV drugs	2.6% of medication was administered at a dose higher or lower than the dose prescribed. Timing error—5.6% exceeding 30 min Administration stage 4% wrong infusion rate
Sheu et al. [17]	IV infusion				328—Actual error (259), Intercepted error (69) Wrong dose 86/259 Wrong drug 81/259 The wrong type of administration 32/259 Wrong patient 30/259 Wrong delivery 22/259
Sorgini et al. [18]	All IV infusion	IV medication doses	NS	62.9	Incomplete labelling of IV tubing (50.1%) Incorrect labelling of IV tubing (42.5%) Inappropriate Y‐site or piggy‐back (6.7%) Inappropriate infusion rate (0.2%) Inappropriate concentration (0.08%) Inappropriate diluent (0.03%)
Prot et al. [19]					Administrative error (excluding timing error): 352 Timing error: 186
Chua et al. [21]	All the drugs (oral and IV)			1114	
Taxis and Barber [22]	35 different IV drugs			430 intravenous doses	155 doses (36%)
Barber and Taxis [23]	All intravenous medication prescription			122 IV Drug doses	22/132
Hermanspann et al. [24]	Injectable drugs	ICU 5040 NICU/PICU 2514		884 preparations ICU‐603 NICU/PICU‐281	Medication error ICU 1/603, NICU 0/281 Type of solution ICU 0/465, NICU 1/253 The volume of solution ICU 22/465, NICU 4/253 Uniform mixing ICU 227/351, NICU 14/226 Reconstitution ICU 8/144, NICU 0/96 Dose ICU 2/603, 0/281 Timing error ICU 0/603, NICU 7/281 Route ICU 0/603, NICU 0/281 Infusion rate ICU 16/603, NICU 9/281
Vijaya Kumar et al. [25]	The patients who received more than two IV medications			110 patients	Error in rate of administration = 12/100 = 10.9%, Dilution error 9/110 = 8%
Westbrook et al. [28]	Any intravenous medication			568 intravenous preparation and administration	Wrong rate = 266 Wrong volume = 121 Wrong mix = 21 Drug incompatibility = 3 Total intravenous administrations with at least one clinical error = 363
Ong and Subasyini [26]	IV drug preparation and administration	Nurses		349	302/349
Wirtz [27]	Any Intravenous medication				93/278 administration errors Wrong administration rate 73 Omissions 36 Wrong dose 34

Abbreviation: NS, not specified.

**Table 3:** Summary of the intravenous medication error and the study score for three quality area.

References (Cross‐Sectional Studies)	Newcastle‐Ottawa Scale									Total score (Maximum 9)			Mean Score of each study
	Selection (Maximum 4)			Comparability (Maximum 2)			Assessment (Maximum 3)						
	R1	R2	R3	R1	R2	R3	R1	R2	R3	R1	R2	R3	
Cousins et al. [7]	3	4	4	0	1	1	1	3	2	4	8	7	6.33
de Castro et al. [10]	2	4	3	0	1	1	2	2	2	4	7	6	5.67
Ding et al. [9]	1	4	4	1	1	1	2	3	2	4	8	7	6.33
Fahimi et al. [11]	2	5	3	1	1	1	2	3	3	5	9	7	7.00
Fahimi et al. [12]	2	4	3	1	1	1	2	3	3	5	8	7	6.67
Fekadu et al. [33]	4	4	4	0	1	0	3	3	3	7	8	7	7.33
Hoefel et al. [13]	3	4	3	0	1	0	2	3	3	5	8	6	6.33
Nguyen et al. [14]	3	4	4	0	1	0	2	3	3	5	8	7	6.67
Basil et al. [35]	2	5	4	1	1	1	2	3	3	5	9	7	7.00
Anselmi et al. [15]	3	4	3	1	1	1	2	3	3	6	8	7	7.00
Dhamija et al. [16]	2	4	4	0	1	1	2	3	2	4	8	7	6.33
Mendes et al. [20]	2	4	3	0	1	0	2	3	3	4	8	6	6.00
Shuh et al. [17]	2	4	3	0	1	1	2	1	2	4	6	6	5.33
Sorgini et al. [18]	3	4	3	1	1	1	2	3	3	6	8	7	7.00
Prot et al. [19]	2	4	3	0	1	1	2	3	2	4	8	6	6.00
Chua et al. [21]	3	5	4	0	1	1	2	2	2	5	8	7	6.67
Taxis and Barber [22]	3	4	3	0	1	1	2	3	3	5	8	7	6.67
Barber and Taxis [23]	3	4	4	0	1	1	2	3	2	5	8	7	6.67
Hermanspann et al. [24]	3	4	3	1	1	1	2	3	2	6	8	6	6.67
Vijaya Kumar et al. [25]	3	4	3	1	1	1	2	3	3	6	8	8	7.33
Westbrook et al. [28]	4	4	4	1	1	1	3	3	3	8	8	8	8.00
Ong and Subasyini [26]	3	4	4	0	1	0	2	3	3	5	8	7	6.67
Wirtz [27]	3	4	3	0	1	0	2	3	3	5	8	6	6.33
Mean score													6.61

## 4 Discussion

### 4.1 Main Findings

Our current systematic review shows a wide range in the prevalence of medication errors. A systematic review of medication errors and error‐related adverse events in patient care settings showed that variation was high due to heterogeneity in population, study design, and outcome evaluated [15]. Although the included studies varied in terms of setting (e.g., teaching hospitals, university hospitals, and Ministry of Health [MOH] institutions), study design, and population, several consistent themes were identified. Day‐to‐day pharmacist involvement (PI) in clinical rounds appeared relatively uniform across these institutions. However, differences were observed in medication procurement practices and formulary restrictions, especially in MOH hospitals, which may limit pharmacists' ability to make individualised medication suggestions. The overrepresentation of studies from teaching hospitals may reflect the academic research emphasis within those institutions. Despite this heterogeneity, the findings offer valuable insights into the nature and frequency of IV medication errors across healthcare settings. The study population and setting vastly affect the medication error rate, similar to other systematic reviews. This systematic review differs from prior reviews by focusing exclusively on primary studies reporting IV medication errors in hospitalised patients. While some review articles were referenced for contextual understanding, our analysis is based on the direct extraction of error rates, contributing factors, and intervention effectiveness from original studies. By synthesising primary data, this review provides a comprehensive and updated perspective that enhances the understanding of IV medication administration errors.

Medication errors are notably prevalent in paediatric and neonatal intensive care settings. While only a minority of included studies specifically investigated neonatal or paediatric settings, they were highlighted in the discussion due to the unique vulnerability of this population. Paediatric and neonatal patients require weight‐based dosing, which introduces complexity and increases the risk of error. Furthermore, pharmacists working in paediatric wards often encounter greater scrutiny in dose calculation and verification, reinforcing the critical nature of their role. The emphasis on these findings serves to underscore important safety considerations rather than overstate their representation in the data set. A systematic review by Alghamdi et al. reported that the median medication error rate in paediatric ICUs was 14.6 per 100 medication orders, with an interquartile range of 5.7%−48.8%. In neonatal ICUs, error rates varied from 4 to 35.1 per 1000 patient days. The most common errors involved prescribing and administration, particularly dosing inaccuracies, with anti‐infective agents frequently implicated [36]. These findings underscore the critical need for targeted interventions to address dosing errors and the use of high‐risk medications in these vulnerable populations. Medication administration errors are highly prevalent due to multiple contributing factors in neonatal ICUs. A nationwide prospective observational study in Malaysia examined 1,093 medication doses, revealing that 743 doses had at least one error, yielding an administration error rate of 68.0%. The most frequent errors were incorrect administration rates (21.2%), improper drug preparation (17.9%), and incorrect dosages (17.0%). Significant factors associated with these errors included IV administration, absence of standardised protocols, a higher number of prescribed medications, increased nursing experience, non‐ventilated neonates, and lower gestational age [37]. These findings highlight the complexity of medication administration in neonatal ICUs and the necessity for standardised protocols and targeted training to reduce errors.

Similarly, in adult ICU settings, IV medication administration errors remain a significant concern. A study by Hermanspann et al. observed 603 IV preparations in an adult ICU, identifying 385 errors, resulting in an overall error rate of 7.6%. Most of these errors pertained to “uniform mixing” during drug preparation. The study highlighted that liquid concentrate dilutions were more frequently associated with errors than other preparation types, emphasising the need for improved nurse training and prefilled syringes to enhance medication safety [24]. Further emphasising the risks associated with IV medication administration, Fekadu et al. reported that the prevalence of IV medication administration errors in hospitalised patients was 46.1%, with missed doses being the most frequent error type. Their study also found that age was a significant risk factor, with higher error rates observed among older adults aged 60–79 years and those over 80 years [33]. This finding aligns with prior research suggesting that elderly patients are particularly vulnerable due to polypharmacy, altered pharmacokinetics, and the complexity of IV medication regimens. Additionally, Henry et al. examined the impact of workload and staffing levels on medication errors, finding that a higher nurse‐to‐patient ratio was significantly associated with increased error rates, particularly in IV drug administration [38]. Their study highlighted the need for optimising nurse workloads and improving support systems to mitigate the risks of medication administration errors in hospital settings.

Most studies in the current systematic review used a single observer (trained nurse or pharmacist) to detect medication errors. One approach to mitigating these errors is the involvement of clinical pharmacists. A study by Simpson et al. demonstrated that a pharmacist‐led education programme, which included daily reviews of medication orders and mandatory dose calculation assessments for new staff, significantly reduced medication errors in neonatal ICUs [39]. This finding reinforces the value of pharmacist participation in medication safety initiatives, particularly in high‐risk settings such as neonatal and paediatric ICUs. Additionally, the direct observation method is more efficient and accurate than reviewing charts and incident reports in detecting medication errors [12]. In data collection using the observation method for medication errors, participants can be affected in several ways. A study conducted on medication error and adverse drug events in the intensive care unit showed that using a direct observation approach, the study found a higher incidence of potential and actual, preventable adverse drug events and an increased ratio of potential to actual, preventable adverse drug events [13]. The Hawthorne effect may cause healthcare professionals to alter their behaviour due to awareness of being observed, potentially reducing errors. These studies suggest that the Hawthorne effect can lead to observational bias, overestimating treatment effectiveness, and misinterpreting target populations in various medical research contexts [14, 16]. Privacy and confidentiality concerns arise for patients and professionals, necessitating strict ethical guidelines and informed consent. Observations can lead to workflow disruptions, increased stress, and potential biases in data interpretation.

Additionally, the presence of observers might cause emotional distress or anxiety about possible repercussions. To mitigate these impacts, it is essential to maintain confidentiality, minimise observer interference, provide clear reassurances about the non‐punitive nature of the study, and ensure informed consent from all participants. Proper training for observers and standardised protocols are crucial for reducing these effects and ensuring accurate and reliable data collection. Studies also stated that observation method observation of nurses during drug administration did not significantly affect the medication error rate, and observer reliability was high [20].

IV medication errors in this study reporting vary widely, with some studies presenting overall error rates while others detail specific types. Accurate reporting is essential for improving patient safety, prioritising preventive strategies, ensuring regulatory compliance, and guiding healthcare policies. Factors contributing to errors include human factors like fatigue and inexperience, communication issues, systemic challenges such as inadequate staffing, and the complexity of multi‐drug therapies [40, 41, 42]. Addressing these issues through enhanced workflow design and staff education can reduce errors [21]. A key challenge in analysing IV medication errors is the inconsistent definitions used across studies—some focus on dose omissions, others on incorrect ingredients, deviations from prescriptions, or only preventable errors. This variability complicates comparisons and limits generalisability. Standardising medication preparation and administration processes, implementing preventive measures, and training staff on proper admixture protocols are essential to reducing errors and enhancing patient safety [7].

### 4.2 Limitations

This study of medication errors in healthcare settings is fraught with challenges, primarily due to heterogeneity in definitions and methodologies. This leads to inconsistent error rates and types reported, making cross‐study comparisons and generalisations difficult. Examples of the diversity of definitions exist for medication errors, the hospital and ward settings, and the lack of denominators for calculating the prevalence of medication errors in certain studies. Many studies were excluded due to predefined eligibility criteria designed to ensure methodological rigor and data reliability. While this may have limited the total number of included studies, excluding those with insufficient or unclear denominator data, methodological weaknesses, or lack of specific focus on IV medication errors was necessary. The diversity in ward settings could highly influence the error rate. For example, the intensive care unit setting has a lower patient‐to‐nurse ratio than general medical wards. A systematic review of interventions to reduce medication error in intensive care units identifies eight types of computerised physician order entry (CPOE), changes in work schedules (CWS), IV systems (IS), modes of education (ME), medication reconciliation (MR), PI, protocols and guidelines (PG) and support systems for clinical decision making (SSCD) [30]. Most systematic reviews focus on specific healthcare settings or patient populations, limiting the generalisability of findings to broader contexts. Additionally, the studies used different methods for detecting medication errors, such as spontaneous reporting, chart reviews, and direct observation, resulting in varied error rates, with self‐reporting and incident reporting systems often underestimating the true incidence. There is also a notable scarcity of systematic reviews evaluating the effectiveness of interventions to reduce medication errors, and those that do exist often suffer from high bias and lack robust study designs. Furthermore, the tools used to assess the severity of prescribing errors lack reliability and validity, complicating the comparison of findings across studies and underscoring the need for standardised, reliable, and valid assessment tools.

### 4.3 Future Research

Research looking into the qualitative and quantitative nature of medication administration errors in healthcare settings found that human factors, training and education, work environment, medication complexity, safety culture, and regulatory policy are much needed [31]. Alzoubi et al. [32] conducted a cross‐sectional study among critical care nurses, revealing varied perceptions of medication administration errors. Nurses expressed concerns about patient safety, workload impact, and organisational support. The study underscores the importance of addressing nurses' perceptions to enhance medication safety practices in critical care settings [32]. Inadequate measures to ensure the safe administration of high‐alert medications, limited understanding of the medication, errors in dosage calculations, lapses in double‐checking procedures, and confusion between medications with similar appearances or names were identified as the primary factors contributing to IV medication errors [4].

The studies on IV medication errors did not assess the severity of the errors, similar to the finding of a systematic review done on IV medication errors in the United Kingdom [5]. The current systematic review is the second systematic review to be conducted on errors related to IV medicine. Mixed‐method multihospital research concluded that medication errors are relatively common in infusion administration; however, most have low potential harm [8]. This study gap on the association between IV medication error and the severity of the error has not been explored well and needs to be studied, too. Besides, there is a need for standardised definitions and classification criteria to improve the comparability and robustness of future research on IV medication errors. Future studies should consider adopting internationally recognised frameworks or consensus guidelines to consistently define and categorise IV medication errors. Additionally, conducting subgroup analyses based on studies with similar methodologies and outcome measures may allow for more precise insights into specific types of IV medication errors and their underlying causes. Establishing a more uniform approach will facilitate a better synthesis of evidence and improve the development of targeted interventions.

## 5 Conclusion

Our systematic review highlights the diverse prevalence of IV medication errors, influenced by study design, setting, and population. The need for standardised definitions, reporting procedures, and reliable tracking methods is evident. Factors such as human elements, system issues, and environmental stressors contribute to medication errors. Future research should focus on improving understanding and addressing these factors to enhance patient safety and healthcare quality.

## Author Contributions

All authors contributed to the study's conception and design. Material preparation, data collection, and analysis were performed by S.R., R.R., S.T., F.b.M.M., and D.S.R. The first draft of the manuscript was written by S.R., and all authors commented on previous versions of the manuscript. All authors read and approved the final manuscript.

## Ethics Statement

Systematic reviews do not require any original research and are not subject to ethics approval.

## Consent

The authors have nothing to report.

## Conflicts of Interest

The authors declare no conflicts of interest.

## Supporting information

Appendix I.

## Data Availability

The data that support the findings of this study are available from the corresponding author upon reasonable request.
