# Supplementary material for: Intravenous Medication Administration Errors in Hospitalised Patients: An Updated Systematic Review
Source: J Eval Clin Pract. 2025 Jun 22;31(4):e70167. doi: 10.1111/jep.70167 (PMC12183495; doi:10.1111/jep.70167)
Supplement: Supplementary file 1 — Appendix I. [file JEP-31-0-s001.docx]

**Appendix I**

**Systematic Review Searches on Different Databases on Medication Error/ Omission in Hospitalised Settings for IV Medication**

**Ovid Medline**

1 medication.mp. [mp=title, book title, abstract, original title, name of substance word, subject heading word, floating sub-heading word, keyword heading word, organism supplementary concept word, protocol supplementary concept word, rare disease supplementary concept word, unique identifier, synonyms, population supplementary concept word, anatomy supplementary concept word]

2 drug.mp. [mp=title, book title, abstract, original title, name of substance word, subject heading word, floating sub-heading word, keyword heading word, organism supplementary concept word, protocol supplementary concept word, rare disease supplementary concept word, unique identifier, synonyms, population supplementary concept word, anatomy supplementary concept word]

1 or 2

4 administration.mp. [mp=title, book title, abstract, original title, name of substance word, subject heading word, floating sub-heading word, keyword heading word, organism supplementary concept word, protocol supplementary concept word, rare disease supplementary concept word, unique identifier, synonyms, population supplementary concept word, anatomy supplementary concept word]

5 error.mp. [mp=title, book title, abstract, original title, name of substance word, subject heading word, floating sub-heading word, keyword heading word, organism supplementary concept word, protocol supplementary concept word, rare disease supplementary concept word, unique identifier, synonyms, population supplementary concept word, anatomy supplementary concept word]

4 and 5

7 ward.mp. [mp=title, book title, abstract, original title, name of substance word, subject heading word, floating sub-heading word, keyword heading word, organism supplementary concept word, protocol supplementary concept word, rare disease supplementary concept word, unique identifier, synonyms, population supplementary concept word, anatomy supplementary concept word]

8 hospital.mp. [mp=title, book title, abstract, original title, name of substance word, subject heading word, floating sub-heading word, keyword heading word, organism supplementary concept word, protocol supplementary concept word, rare disease supplementary concept word, unique identifier, synonyms, population supplementary concept word, anatomy supplementary concept word]

9 inpatient.mp. [mp=title, book title, abstract, original title, name of substance word, subject heading word, floating sub-heading word, keyword heading word, organism supplementary concept word, protocol supplementary concept word, rare disease supplementary concept word, unique identifier, synonyms, population supplementary concept word, anatomy supplementary concept word]

7 or 8 or 9

3 and 6 and 10

Total search results: - 1513

If included in intravenous OR IV: - 229

**Pubmed**

(medication OR drug) AND (administration AND error) AND (ward OR hospital OR inpatient)

Result: - 21396

If included in Intravenous OR IV: - 1519

**CINAHL**

(medication OR drug) AND (administration AND error) AND (ward OR hospital OR inpatient)

Result:- 2741

If included in Intravenous OR IV: - 429
